# Association between genetic variants and esophageal cancer risk

**DOI:** 10.18632/oncotarget.17006

**Published:** 2017-04-10

**Authors:** Chenli Yue, Miao Li, Chenxing Da, Hongtao Meng, Shaomin Lv, Xinhan Zhao

**Affiliations:** ^1^ Department of Internal Medicine Oncology, The First Affiliated Hospital of Xi’an Jiaotong University, Xi’an, Shaanxi 710061, China; ^2^ Department of Respiratory Medicine, Shaanxi Provincial Crops Hospital of Chinese People's Armed Police Force, Xi’an, Shaanxi 710054, China; ^3^ Department of Internal Medicine Oncology, The Fifth People's Hospital of Qinghai Province, Xining, Qinghai 810007, China; ^4^ Department of Gastroenterology, Shaanxi Provincial Crops Hospital of Chinese People's Armed Police Force, Xi’an, Shaanxi 710054, China; ^5^ Medical Department, Shaanxi Provincial Crops Hospital of Chinese People's Armed Police Force, Xi’an, Shaanxi 710054, China

**Keywords:** esophageal cancer, genetic polymorphism, NAF1, han chinese, case-control

## Abstract

We investigated whether single nucleotide polymorphisms (SNPs) in the nuclear assembly factor 1 (*NAF1*) and TNFAIP3-interacting protein 1 (*TNIP1*) gene were associated with susceptibility to esophageal cancer in a Chinese Han population. Five SNPs were genotyped and their relationship with esophageal cancer risk was analyzed in a sample of 386 esophageal cancer patients and 495 unrelated healthy controls recruited from the First Affiliated Hospital of Xi’an Jiaotong University. Patients with the AG genotype of rs2320615 were at lower risk of developing esophageal cancer than those with the GG genotype (adjusted odds ratio [OR] = 0.64, 95% confidence interval [CI] = 0.46-0.90, *P* = 0.009). The rs2320615 SNP was found to be associated with a decreased the risk of esophageal cancer in the dominant model (adjusted OR = 0.70, 95% CI = 0.51-0.96, *P* = 0.026). These results provide the first evidence that the rs2320615 in *NAF1* was associated with reduced risk of esophageal cancer. Further studies with larger samples are warranted to confirm our findings.

## INTRODUCTION

Esophageal cancer is the eighth most common cancer and the sixth leading cause of cancer-related mortality worldwide because of its extremely aggressive nature and the poor survival rate of affected patients [1, 2]. The risk of esophageal cancer has been associated with various environmental and genetic factors, as well as their interactions. Epidemiological evidence indicates that the most important environmental risk factors for esophageal cancer development include cigarette smoking, alcohol drinking, malnutrition, inadequate intake of fruits and fresh vegetables, and frequent consumption of pickled vegetables [3, 4]. However, not all people exposed to these hazards eventually suffer from esophageal cancer. Recent studies have suggested the importance of genetic polymorphisms in the carcinogenesis and development of esophageal cancer [5, 6].

Previous studies identified the nuclear-assembly factor 1 (NAF1) protein is an essential H/ACA assembly factor, which might have a central role in coupling H/ACA snoRNP assembly interact with the carboxy terminal domain (CTD) of RNA polymerase (pol) II, and could facilitate recruitment and binding of the H/ACA core proteins to the nascent snoRNA sequences [7, 8]. In addition, a genome-wide meta-analysis found that one SNP (rs7675998) in *NAF1* was associated with telomere length (*P* = 4.35×10^−16^) [9]. Rs2320615 was reported to be the best surrogates for rs7675998 (pairwise r^2^ = 0.89) [10] and which was found to be associated with longer TL (P-trend = 3.3×10^-4^) [11]. Interestingly, short telomere lengths have been associated with esophageal carcinogenesis [12].

*TNIP1* (TNFα-induced protein 3-interacting protein 1), alternatively called *ABIN-1* (A20-binding inhibitor of NF-κB activation) or *Naf1* (Nef-associated factor 1). TNIP1 is a human cellular protein initially identified as interacting with the HIV proteins nef [13] and matrix [14]. It has been reported that *TNIP1* are associated with some diseases, such as leukemia-lymphoma [15], systemic lupus erythematosus [16–18], psoriatic arthritis [19], systemic sclerosis [20, 21], asthma [22], and gastric carcinoma [23]. A previous study revealed that *TNIP1* inhibited the activation of nuclear factor kappa-B (NF-κB) [24], and another study demonstrated that blocking the NF-κB signaling pathway inhibited esophageal cancer proliferation and metastasis [25].

Given the importance of telomere length and the NF-κB signaling pathway in the development and maintenance of esophageal cancer, we hypothesized genetic polymorphisms in *NAF1* and *TNIP1* may influence esophageal cancer susceptibility. To test this hypothesis, we designed a case-control study including 386 esophageal cancer patients and 495 healthy controls to investigate the potential associations between SNPs and the risk of esophageal cancer in a Han Chinese population.

## RESULTS

The basic characteristics of the cases and controls included in this study are summarized in Table 1. In total, 386 esophageal cancer patients (308 males and 78 females) with a mean age of 60.9 (±9.0) years and 495 healthy controls (180 males and 315 females) with a mean age of 54.5 (±9.4) years were enrolled in our study. The age and gender distributions differed significantly between the cases and healthy controls (*P* < 0.001). To eliminate any residual confounding effects, we adjusted for the variables of age and gender in the subsequent multivariable unconditional logistic regression analysis.

**Table 1:** Characteristics of esophageal cancer cases and healthy controls

Variable	Case N (%)	Control N (%)	*P*
Total	386	495	
Gender			**< 0.001**
Male	308 (79.8)	180 (36.4)	
Female	78 (20.2)	315 (63.6)	
Age,			**< 0.001**
yrs (Mean ± SD)	60.9±9.0	54.5±9.4	

*P* values were calculated with Pearson's χ^2^ test (gender) and Welch's t test (age).

*P* < 0.05 indicates statistical significance.

The five SNPs (rs2320615, rs3792792, rs4958881, rs7708392, and rs10036748) were successfully genotyped in the esophageal cancer cases and healthy controls. The average SNP call rate was 98.3% in cases and controls. The sequences of primers for each SNP polymerase chain reaction (PCR) and single base extension reaction are listed in Table 2. The minor allele frequencies for the five SNPs in the patient samples and normal controls are shown in Table 3. We used a χ^2^ test to compare the differences in the frequency distributions of alleles between cases and controls, but no significant associations between the alleles of the five SNPs were detected. One SNP (rs4958881) was excluded from further analyses because it deviated from Hardy-Weinberg equilibrium in controls (*P* < 0.001).

**Table 2:** Sequences of oligonucleotide primers used to analyze *NAF1* polymorphisms

SNP-ID	1st-PCRP	2nd-PCRP	UEP
rs2320615	ACGTTGGATGACCAATTTAACAAGACAGC	ACGTTGGATGAGGCAGAGACATTCCATTTG	CCATTTGAAAAGAAATAATTCTACT
rs3792792	ACGTTGGATGCTCAGATCAGTTCACTCCTC	ACGTTGGATGATGGCAGCTGTTACGGCCAC	ccctTTACGGCCACCACCAAGCATG
rs4958881	ACGTTGGATGCACAAATATGTGGACAGTTT	ACGTTGGATGTGCAATTCCACCCAAGGATG	GGATGAAAGGAAGTGAGA
rs7708392	ACGTTGGATGAGGCCAACTGGTCAATTCTC	ACGTTGGATGGGGTCTCTTCTGGAACTTAG	ggggaTGGAACTTAGTAGACTAGTCA
rs10036748	ACGTTGGATGGCAAAGCAGCCCCTTTTTTC	ACGTTGGATGCTTTCATAGCATGATACACG	ACGTATGAGAAAAATAAAATAGTAA

SNP: Single nucleotide polymorphism; PCRP: Polymerase chain reaction primer; UEP: unique base extension primer

Sequences are written in the 5’→3’ (left to right) orientation.

**Table 3:** Allele frequencies in cases and controls and odds ratio estimates for esophageal cancer

SNP-ID	Gene	Chr	Band	Position	Role	Alleles (A/B)	MAF		HWE	OR	95% CI	*P*
							Case	Control				
rs2320615	*NAF1*	4	4q32.2	164069949	Intron	A/G	0.178	0.210	0.344	0.81	0.64-1.03	0.090
rs3792792	*TNIP1*	5	5q33.1	150440506	Intron	C/T	0.078	0.063	0.711	1.26	0.87-1.82	0.216
rs4958881	*TNIP1*	5	5q33.1	150450236	Intron	C/T	0.123	0.092	**< 0.001**	1.39	1.02-1.89	0.035
rs7708392	*TNIP1*	5	5q33.1	150457485	Intron	G/C	0.231	0.224	0.438	1.04	0.83-1.30	0.753
rs10036748	*TNIP1*	5	5q33.1	150458146	Intron	C/T	0.229	0.226	0.306	1.02	0.81-1.28	0.864

SNP: Single nucleotide polymorphism; Chr: Chromosome; MAF: Minor allele frequency; HWE: Hardy-Weinberg equilibrium; OR: Odds ratio; 95% CI: 95% Confidence interval.

*P* values were calculated with the Chi-Square test.

*P* < 0.05 indicates statistical significance.

The results of genetic model analyses are shown in Table 4. The AG genotype of rs2320615 was associated with significantly lower risk of esophageal cancer than the GG genotype, both before and after adjustment for age and gender (OR = 0.67, 95% CI: 0.50-0.90, *P* = 0.008; adjusted OR = 0.64, 95% CI: 0.46-0.90, *P* = 0.009, respectively). Furthermore, the SNP (rs2320615) was significantly associated with esophageal cancer risk under the dominant model after adjustment for age and gender (adjusted OR = 0.70, 95% CI: 0.51-0.96, *P* = 0.026). However, no significant association was found between *TNIP1* polymorphisms and esophageal cancer risk.

**Table 4:** Genetic model analyses of the associations between the SNPs and esophageal cancer susceptibility

SNP-ID	Model	Genotype	Case (N)	Control (N)	Without adjustment			With adjustment		
					OR	95% CI	*P*	OR	95% CI	*P*
rs2320615	Co-dominant	GG	263	305	1.00			1.00		
		AG	99	172	0.67	0.50-0.90	**0.008**	0.64	0.46-0.90	**0.009**
		AA	18	18	1.16	0.59-2.28	0.667	1.27	0.58-2.78	0.544
	Dominant	GG	263	305	1.00			1.00		
		AG-AA	117	190	0.71	0.54-0.95	**0.020**	0.7	0.51-0.96	**0.026**
	Recessive	GG-AG	362	477	1.00			1.00		
		AA	18	18	1.32	0.68-2.57	0.418	1.47	0.68-3.19	0.330
	Additive	---	---	---	0.81	0.64-1.04	0.093	0.81	0.62-1.06	0.120
rs3792792	Co-dominant	TT	328	434	1.00			1.00		
		CT	56	60	1.24	0.84-1.83	0.291	1.37	0.88-2.13	0.169
		CC	2	1	2.65	0.24-29.31	0.428	1.79	0.16-20.09	0.636
	Dominant	TT	328	434	1.00			1.00		
		CT-CC	58	61	1.26	0.85-1.85	0.245	1.38	0.89-2.14	0.153
	Recessive	TT-CT	384	494	1.00			1.00		
		CC	2	1	2.57	0.23-28.48	0.441	1.72	0.15-19.26	0.660
	Additive	---	---	---	1.27	0.87-1.84	0.212	1.36	0.90-2.08	0.149
rs7708392	Co-dominant	CC	231	301	1.00			1.00		
		GC	132	166	1.04	0.78-1.38	0.808	1.05	0.76-1.44	0.790
		GG	23	28	1.07	0.60-1.91	0.818	1.22	0.63-2.36	0.553
	Dominant	CC	231	301	1.00			1.00		
		GC-GG	155	194	1.04	0.79-1.37	0.772	1.07	0.79-1.45	0.673
	Recessive	CC-GC	363	467	1.00			1.00		
		GG	23	28	1.06	0.60-1.87	0.849	1.2	0.63-2.30	0.578
	Additive	---	---	---	1.04	0.83-1.29	0.757	1.07	0.84-1.38	0.577
rs10036748	Co-dominant	TT	230	300	1.00			1.00		
		CT	132	165	1.04	0.78-1.39	0.771	1.06	0.77-1.47	0.707
		CC	22	29	0.99	0.55-1.77	0.972	1.11	0.57-2.15	0.763
	Dominant	TT	230	300	1.00			1.00		
		CT-CC	154	194	1.04	0.79-1.36	0.802	1.07	0.79-1.46	0.667
	Recessive	TT-CT	362	465	1.00			1.00		
		CC	22	29	0.97	0.55-1.73	0.929	1.08	0.56-2.08	0.811
	Additive	---	---	---	1.02	0.82-1.27	0.866	1.06	0.82-1.36	0.658

SNP: Single nucleotide polymorphism; OR: Odds ratio; 95% CI: 95% Confidence interval

The logistic regression analysis was adjusted for age and gender.

*P* values were calculated with the Wald test.

*P* < 0.05 indicates statistical significance.

**Figure 1:**
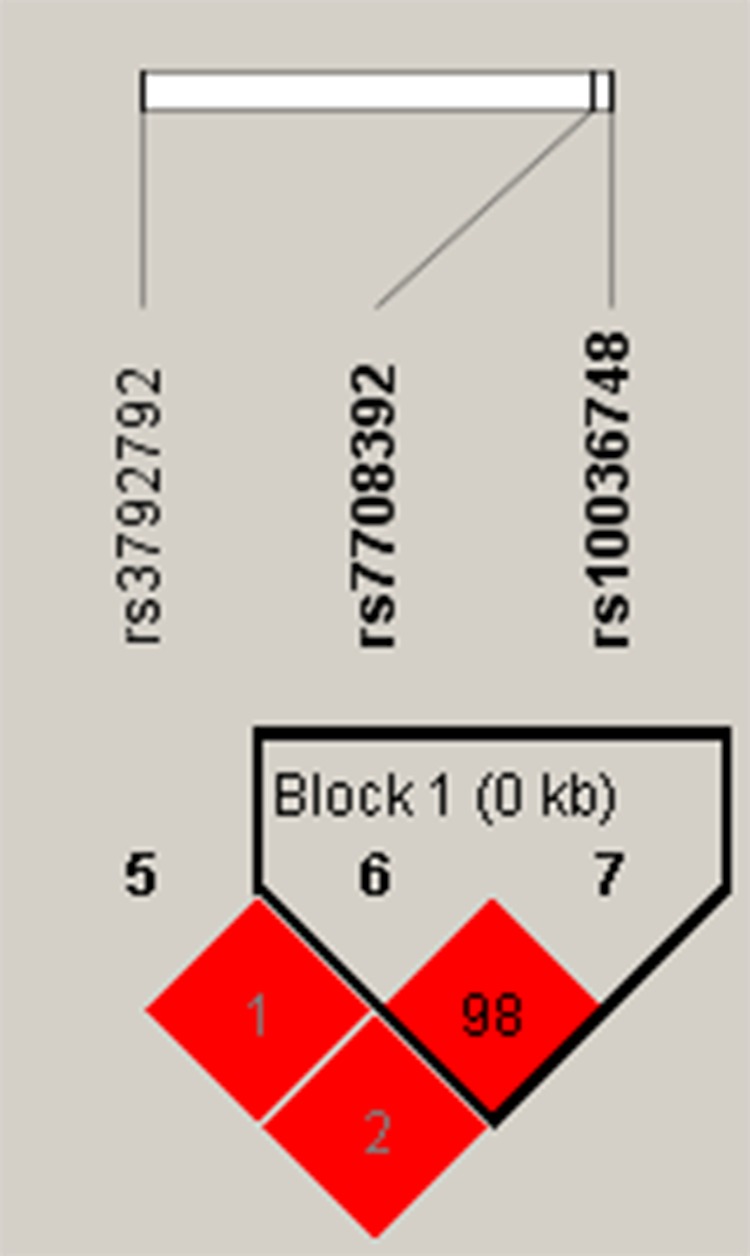
Haplotype block map for the three SNPs in the *TNIP1* gene The LD between two SNPs is expressed as a standardized D’ value (red schemes).

As shown in Figure 1, we conducted linkage disequilibrium analysis for the four SNPs, and one linkage disequilibrium block consisted of two SNPs (rs7708392 and rs10036748) which exhibited statistically significant linkage (r^2^ = 0.98).

## DISCUSSION

This case-control study was designed to investigate whether genetic variants of *NAF1* and *TNIP1* were associated with the risk of esophageal cancer in the Han Chinese population. Our data demonstrated that the rs2320615 in *NAF1* was associated with significantly reduced risk of esophageal cancer.

*NAF1* encodes a protein important for the assembly of H/ACA human telomerase RNA [8]. This chaperone protein is involved in the formation and activity of telomerase, and a previous study indicated that *NAF1* polymorphism (rs7675998) is associated with mean telomere length [9]. Interestingly, short leukocyte telomere lengths are associated with significantly increased risk of developing esophageal cancer [12]. Our results suggested that *NAF1* polymorphisms are associated with the risk of esophageal cancer. However, it remains unclear whether these *NAF1* polymorphisms affect telomere length by affecting specific protein synthesis and function, and ultimately the susceptibility to esophageal cancer. This assumption will be tested in future gene functional experiments.

Our results demonstrated that the genotype AG of rs2320615 in *NAF1* was associated with a significantly reduced risk of esophageal cancer. A study found that the rs2320615 is associated with longer telomere length [11]. Because we did not explore the association of the SNP with telomere length and the association between telomere length and esophageal cancer risk in this study, it is unclear whether these SNPs affect telomere length, which in turn affects the risk of esophageal cancer. It is possible that polymorphisms can affect the expression of genes and thus render individuals susceptible to esophageal cancer. We will confirm our results and speculation in future studies.

Two recent GWASs revealed that rs7708392 and rs10036748 were associated with the risk of systemic lupus erythematosus [16, 17]. A previous report also indicated that the G allele of rs7708392 and the C allele of rs10036748 were associated with increased risk of gastric carcinoma, and that the haplotype CT (rs7708392-rs10036748) was associated with a reduced risk of gastric carcinoma [23]. In addition, a significantly decreased association was found between the allele T of rs10036748 and asthma risk [22]. Recently, the NF-κB signaling pathway was reported to be associated with tumorigenesis in esophageal cancer [25]. NF-κB is a ubiquitous transcription factor that involves in immunity and inflammation, regulates cell proliferation, apoptosis and migration, and is constitutively activated in a number of human cancers, including some esophageal cancers [26–28]. Previous studies have demonstrated that *TNIP1* can inhibit NF-κB activity [29, 30]. However, no significant association between *TNIP1* and esophageal cancer risk was found in the study.

There are some limitations to the present case-control study. First, the relatively small sample did not have sufficient statistical power to reflect the precise association between SNPs and the susceptibility to esophageal cancer. Second, many other risk factors (e.g., smoking, alcohol drinking) were not included due to a lack of corresponding clinical information. Third, biological function analyses were not conducted and deserve further study.

In conclusion, the current study has identified that rs2320615 in the *NAF1* was associated with the risk of esophageal cancer in the Han Chinese population. These findings provide a theoretical foundation for further research (with a large sample) into the association between *NAF1* and *TNIP1* and esophageal cancer risk in other populations.

## MATERIALS AND METHODS

### Study subjects

We recruited 386 patients who were diagnosed with esophageal cancer at the First Affiliated Hospital of Xi’an Jiaotong University between July 2014 and October 2015. The 495 controls without histories of cancer or other major diseases were randomly selected from the health evaluation center at the same hospital during the same time period. All participants were of ethnic Han origin, and their ancestors had lived in the region for at least three generations. Patients who previously had cancer, radiotherapy or chemotherapy were excluded. All cases were recruited without any restrictions regarding age, sex, or disease stage. In addition, each subject was personally questioned by trained interviewers, who used a pre-tested questionnaire to obtain basic information, including demographic data (e.g., age, sex) and some clinical information.

This study was approved by the Ethical Committee of the First Affiliated Hospital of Xi’an Jiaotong University, and complied with the World Medical Association Declaration of Helsinki. All individuals gave written informed consent to be included in the study.

### DNA extraction

A vacutainer was used to collect a peripheral venous blood sample (5 mL) fromeach study subject, and the sample was transferred to a tube containing ethylenediamine tetra-acetic acid. All blood samples were stored at -20°C until DNA extraction. Genomic DNA was isolated from whole blood with the GoldMag-Mini Whole Blood Genomic DNA Purification Kit according to the manufacturer's protocol (GoldMag. Co. Ltd., Xi’an, China). The concentration of the isolated DNA was measured with a spectrophotometer (NanoDrop 2000; Thermo Fisher Scientific, Waltham, MA, USA) at wavelengths of A260 and A280 nm. The DNA was diluted with QIAgility to a final concentration of 20 ng/μl.

### SNP selection and genotyping

Five SNPs (rs2320615, rs3792792, rs4958881, rs7708392 and rs10036748) with had minor allele frequency > 5% in the HapMap of the Chinese Han Beijing population that have been reported to be associated with the risk of several diseases and cancers, including systemic lupus erythematosus [16–18], systemic sclerosis [21], asthma [22], and gastric carcinoma [23]. They were selected for further genotyping. Primers for the amplification process and single base extension reactions were designed with Sequenom Mass-ARRAY Assay Design 3.0 Software (Sequenom, San Diego, CA, USA). Genotyping of the SNPs was performed with the Sequenom MassARRAY platform [31] (Sequenom, San Diego, CA, USA) according to the standard instructions recommended by the manufacturer. Sequenom Typer 4.0 software was used for data management and analyses.

### Statistical analysis

For cases and controls, we analyzed the gender distribution using Pearson's χ^2^ test and the age distribution by Welch's t test. We analyzed the genotype frequencies in the controls with Fisher's exact test to determine whether the five SNPs departed from Hardy-Weinberg equilibrium. The differences in the SNP allele and genotype distributions between patients and controls were evaluated with Chi-squared test/Fisher's exact tests. Genetic model analyses (Dominant, Recessive and Additive) were performed with PLINK software to assess the significance of the SNPs. The associations of the SNPs with the risk of esophageal cancer were estimated from the odds ratios (ORs) and 95% confidence intervals (CIs), which were determined by unconditional logistic regression and adjusted for age and gender [32]. The *P*-values reported are two-sided, and values of *P* < 0.05 were considered to be statistically significant. We used the Haploview software package (version 4.2) platform for analyses of pairwise linkage disequilibrium (LD) and haplotype structure [10]. All statistical analyses were performed with Microsoft Excel and SPSS 19.0 (SPSS, Chicago, IL, USA).
